# Tumour stromal cells derived from paediatric malignancies display MSC-like properties and impair NK cell cytotoxicity

**DOI:** 10.1186/1471-2407-10-501

**Published:** 2010-09-21

**Authors:** Pascal-David Johann, Martin Vaegler, Friederike Gieseke, Philippa Mang, Sorin Armeanu-Ebinger, Torsten Kluba, Rupert Handgretinger, Ingo Müller

**Affiliations:** 1 University Children's Hospital, Department of General Paediatrics, Hematology and Oncology, Tübingen, Germany; 2 University Children's Hospital, Department of Paediatric Surgery, Hematology and Oncology, Tübingen, Germany; 3 University Hospital Tübingen, Department of Orthopedics, Tübingen, Germany; 4 Clinic of Paediatric Haematology and Oncology, University Medical Center Hamburg-Eppendorf, Hamburg, Germany

## Abstract

**Background:**

Tumour growth and metastatic infiltration are favoured by several components of the tumour microenvironment. Bone marrow-derived multipotent mesenchymal stromal cells (MSC) are known to contribute to the tumour stroma. When isolated from healthy bone marrow, MSC exert potent antiproliferative effects on immune effector cells. Due to phenotypic and morphological similarities of MSC and tumour stromal cells (TStrC), we speculated that immunotherapeutic approaches may be hampered if TStrC may still exhibit immunomodulatory properties of MSC.

**Methods:**

In order to compare immunomodulatory properties of MSC and tumour stromal cells (TStrC), we established and analyzed TStrC cultures from eleven paediatric tumours and MSC preparations from bone marrow aspirates. Immunophenotyping, proliferation assays and NK cell cytotoxicity assays were employed to address the issue.

**Results:**

While TStrC differed from MSC in terms of plasticity, they shared surface expression of CD105, CD73 and other markers used for MSC characterization. Furthermore, TStrC displayed a strong antiproliferative effect on peripheral blood mononuclear cells (PBMC) in coculture experiments similar to MSC. NK cell cytotoxicity was significantly impaired after co-culture with TStrC and expression of the activating NK cell receptors NKp44 and NKp46 was reduced.

**Conclusions:**

Our data show that TStrC and MSC share important phenotypic and functional characteristics. The inhibitory effect of TStrC on PBMC and especially on NK cells may facilitate the immune evasion of paediatric tumours.

## Background

Solid tumours are composed of tumour stromal cells, blood vessels, infiltrating immune cells and tumour cells themselves. Over the last decade, a growing body of literature has highlighted the importance of the tumour microenvironment for the prognosis of different types of cancer [1]. The significance of tumour stroma for the overall prognosis may be in part due to the fact that several components of the tumour-microenvironment have been shown to compromise immune effector functions against tumour cells [2]. Tumour invading immune cells are functionally impaired within tumours: NK cells, derived from non small cell lung tumours display a decreased cytotoxicity against cancer cells *in vitro *and differ from NK cells from peripheral blood not only by a different cytokine secretion, but also by other functional alterations [3]. In a comprehensive study, tumour-infiltrating lymphocytes were analysed and regulatory T cells could be identified in all tumour samples, which impair anti-tumour responses of immune effector cells [4]. More evidence for the immunological activities of tumour stroma came from the elimination of cancer associated fibroblasts in a murine breast cancer model resulting in a shift from Th2 to Th1 polarization [5]. Hence, tumour stromal cells (TStrC) may participate in regulation of immune effector functions at several levels [6]. However, the exact mechanisms are poorly understood. The site of origin and recruitment of TStrC into the tumour have been identified as key issues in the elucidation of TStrC function in the microenvironment [7]. TStrC resemble multipotent mesenchymal stromal cells (MSC) in morphological aspects and MSC might indeed be a source for these specialized stromal cells [8]. MSC have been shown to suppress proliferation and alloreactivity of T cells [9-11]. Furthermore, they modulate functions of B cells and of dendritic cells [12] and, importantly, MSC do not only inhibit the proliferation of NK cells but also suppress their cytotoxic activity [13-15]. These immunological properties may contribute to tumour spread as MSC can be found in human breast cancers and promote metastasis [16]. Bioluminescence imaging of mice indicated a tropism of bone marrow-derived MSC to inflammatory microenvironments such as tumours [17]. In this context, the inhibitory effects of MSC on virtually all cells of the immune system may be relevant [12]. To investigate immunological features of stromal cells in neuroblastomas and other paediatric tumours, we isolated TStrC and hypothesized that immunomodulatory properties of these cells may contribute to the immune evasion of tumours. When we focused on NK cells, we found that the activating NK cell receptors NKp44 and NKp46 were downregulated while the inhibitory receptor NKG2A remained unaffected. This may be one mechanism to inhibit lysis of e. g. neuroblastoma cells, which are known to express only low densities of HLA molecules and represent good NK cell targets [18].

## Methods

### Cell culture and isolation of tumour stromal cells

Excessive material after pathological analysis served as starting material (Table 1). Informed written consent was obtained from the parents and the protocol approved by the local IRB (892007V). Histological diagnosis was confirmed by the Institute of Pathology, University of Tübingen. Tumour tissue was disrupted mechanically and placed in 2 ml DMEM medium low-glucose (LG-DMEM, Lonza, Basel, Switzerland), supplemented with 5% (v/v) human fresh frozen plasma (FFP), 10^7^/mL platelets (University of Tübingen blood donor center), 80 IU/mL heparin sulphate (Medunasal, Isernhagen, Germany), 100 IU/mL penicillin and 100 mg/mL streptomycin (Biochrom, Berlin, Germany), 2 mM glutamine (Biochrom) and incubated at 37°C under a water saturated atmosphere with 10% CO_2_. After 7-9 days, first TStrC colonies appeared. Non-adherent cells were washed away and adherent cells were detached using trypsin (Lonza) when confluency of 80% was reached. Cells were re-plated at a density of 2000 cells/cm^2 ^in tissue culture flasks. Cell cultures, which were employed for experiments, did not exceed a number of twelve passages. All tumour samples except for sample no. 6 were obtained after chemotherapy. Isolation efficiency of TStrC did not vary considerably between different tumour samples. The formation of first TStrC colonies and the propagation of the cells took longer compared to bone-marrow derived MSC.

**Table 1:** TStrC were isolated and propagated from eleven patients.

Sample number	Tumour	Age at resection	Chemotherapy prior to surgery
1	Neuroblastoma	2 years	NB04 Trial Protocol

2	Neuroblastoma	11 years	NB04 Trial Protocol

3	Neuroblastoma	4 months	NB04 Trial Protocol

4	Neuroblastoma	4 years	NB04 Trial Protocol

5	Neuroblastoma	3 months	NB04 Trial Protocol

6	Teratoma	1 month	No prior treatment

7	Osteosarcoma	17 years	COSS96 Trial Protocol

8	Osteosarcoma	20 years	Euramos 1 Trial Protocol

9	Ewing sarcoma	16 years	Euro-Ewing 99 Trial Protocol

10	Rhabdomyosarcoma	16 years	CWS IV 2004 Trial Protocol

11	Rhabdomyosarcoma	8 years	CWS IV 2004 Trial Protocol

The acute myeloid leukaemia cell line K562 was obtained from ATCC (Wesel, Germany) and cultured under standard conditions. Isolation and culture of bone marrow-derived MSC from paediatric patients with haematologic disorders were performed as described previously [19].

### Differentiation Assays

Differentiation assays of TStrC and MSC towards the osteogenic and adipogenic lineages were performed as described earlier [20]. Briefly, cells were seeded into LG-DMEM Medium containing 5% FFP, 80 IU/mL heparin sulphate, 100 IU/mL penicillin and 100 mg/mL streptomycin, glutamine (2 mM), supplemented with dexamethasone (10 nM), L-ascorbic acid-2-phosphate (0.1 mM), beta-glycerol phosphate (5 mM) (all Sigma, Munich, Germany) and BMP-2 (100 ng/ml) (Tebu-Bio, Magenta, Italy). After 14-21 days, differentiated stromal cells and controls were stained with aqueous 0.5% (v/v) Alizarin Red-S (Sigma) and washed with PBS.

For adipogenic induction, MSC and TStrC were plated in LG-DMEM supplemented with 5% FFP, 80 IU/mL heparin sulphate, 100 IU/mL penicillin and 100 mg/mL streptomycin, glutamine (2 mM), 1 mM dexamethasone, 60 nM indomethacin, 10 mM rh-insulin and 0.5 mM isobuthylmethylxanthine. After 14-21 days differentiation was verified by Oil-Red-O (Sigma) staining.

### Immunophenotyping

Flow cytometric analysis was performed on a FACS Calibur (Becton Dickinson) and data was analyzed by the CellQuestPro software, Version 4.0.2. (Becton Dickinson). Anti-IgG1-FITC (clone MOPC-31C), anti-IgG1-PE (clone G18-145), anti CD45-FITC (HI30), anti-CD34-PE (563), anti-CD56-FITC (B159), anti-CD73-PE (A02), anti-HLA-DR-FITC (TÜ36) anti-HLA-ABC-PE (G46-2.6), anti-NKp44 (P44-8.1), anti-NKp46 (9E2/NKp46), anti-CD69 (FN50) and anti-NKG2A (20d5) monoclonal antibodies were obtained from Becton Dickinson; additionally, anti-CD105-FITC (N1-3A1) was purchased from Ancell, Bayport, MN (USA). Staining of the cells was performed as described previously [20].

### CFSE Assays

Proliferation of PBMC was determined using the CFSE assay as described earlier [20]. Briefly, 75,000 HLA mismatched PBMC were added to each well of a 96-well plate, already containing 5,000, 10,000, 20,000 or 30,000 TStrC, respectively. IL-2 (BD Biosciences) and OKT3 (Janssen-Cilag, Neuss, Germany) were used as stimuli where indicated. For coculture experiments under hypoxic conditions the plate was placed into a HERA cell incubator (Heraeus Instruments GmbH, Hanau, Germany) at 1% O_2 _and 10% CO_2_.

### Isolation of NK cells and coculture experiments

NK cells were isolated by immunomagnetic selection with CD56^+ ^magnetic beads (Miltenyi Biotech, Bergisch Gladbach, Germany) from PBMC of healthy donors (IRB approval 892007V). Purity of isolated NK cells was controlled by staining for CD56 after isolation and exceeded 95% for NK cells that were employed in experiments. For CFSE-proliferation experiments, 75,000 CFSE-stained NK cells were added into each well of a 96-well plate, already containing 10,000, 20,000 or 30,000 TStrC, respectively. Stimulation of NK cells was performed using 100 IU/ml IL-2. Phenotyping of NK-cells after coculture of four days was performed by flow cytometry with the antibodies described above.

### Cytotoxicity assay

The effect of TStrC and MSC on NK cell function was assessed by the BATDA cytotoxicity assay as described previously [21]. NK cells were cocultured with TStrC for four days at a ratio of 4:1 in presence of 100 U/ml IL-2 where indicated. NK cells cultured with 100 U/ml IL-2 served as a control. After the coculture period of 4 days, NK cells were removed from the stroma. Cytotoxicity of NK cells against the leukaemia cell line K562 as standard target was tested in triplicates. Effector cells and target cells were incubated for 2 hours.

### Statistics and data analysis

**Figure 1:**
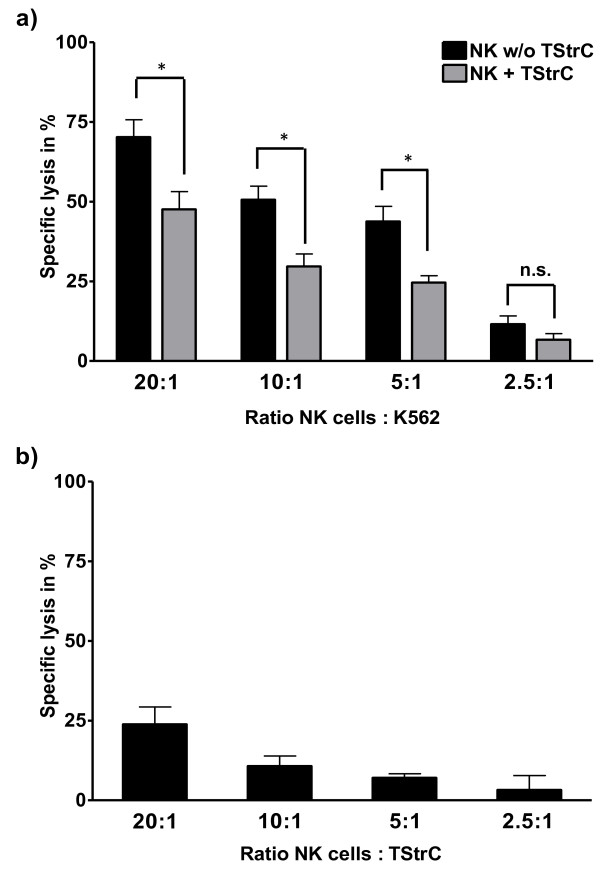
**TStrC-mediated suppression of cytotoxic activity of NK cells**. a) NK cells activated with 100 IU/ml IL-2 displayed a reduced cytotoxicity against K562 (p < 0,05) at NK cell to K562 ratios of 20:1, 10:1 and 5:1, when the NK cells were cocultured for four days with the TStrC (grey bars) in presence of 100 IU/ml IL-2 prior to the cytotoxicity assay; for control, NK cells were cultured alone in the presence of 100 IU/ml IL-2 for four days (black bars). (n = 5). b) NK cells displayed low cytotoxicity against TStrC themselves after stimulation with 100 IU/ml IL-2 for 18 hours (n = 3).

**Figure 2:**
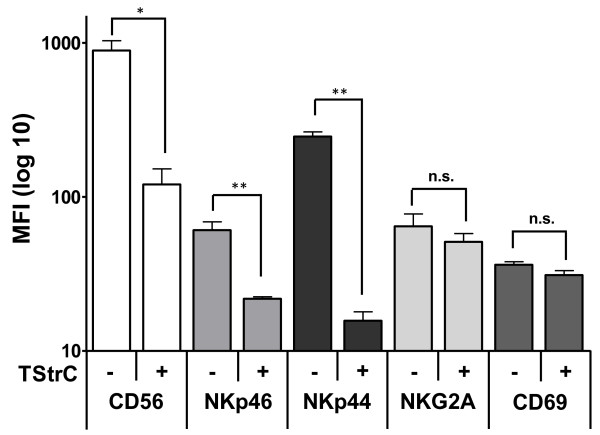
**TStrC modulate immunophenotype of NK cells**. Phenotyping of NK cells cocultured with TStrC for four days in the presence of 100 U/ml IL-2. For control IL-2 stimulated NK cells were cultured without TStrC for four days. The cocultured NK cells displayed a significantly reduced expression of the activating receptors NKp44 and NKp46 (p < 0,01), as well as CD56 (p < 0,05). Remarkably, the expression of the activation marker CD69 and of the inhibitory receptor NKG2A remained unaffected (n = 3).

For figures showing mean values and standard deviation, experiments were performed three time and samples were analysed in triplicates. Statistical analysis of Figure 1a and Figure 2 was performed with student's t-test (one-tailed, unpaired) using Microsoft Excel software (Windows, Redmond, WA). p-values < 0.05 were considered as statistically significant (indicated by an asterisk), p-values < 0.01 as highly significant (indicated by double asterisks).

## Results

### Establishment and characterisation of primary tumour-stromal cell cultures

**Figure 3:**
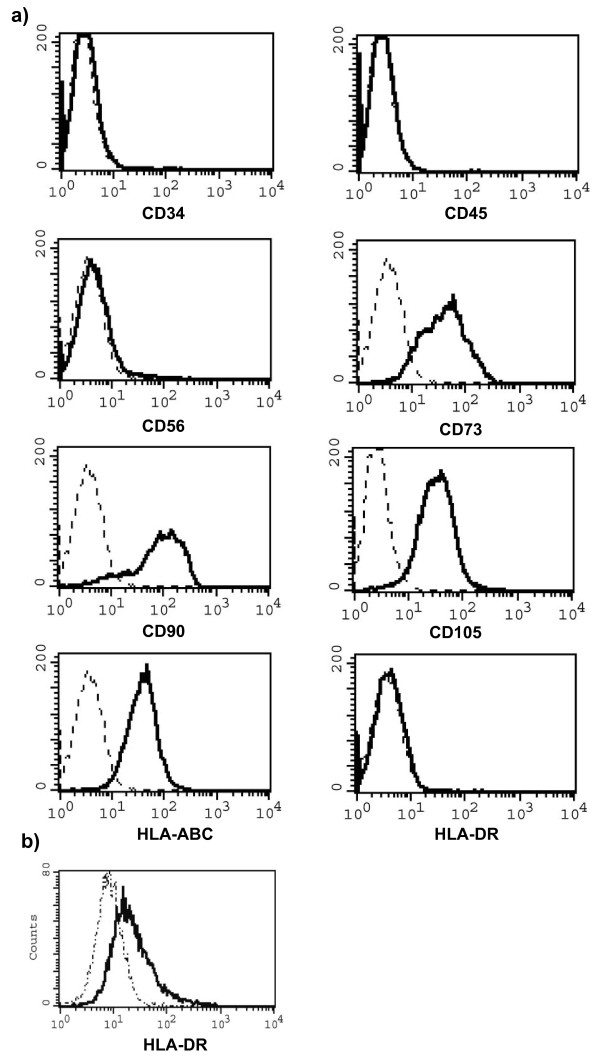
**Immunophenotype of TStrC**. TStrC displayed a MSC-like cell surface marker profile with negativity for markers of the hematopoietic system (CD34, CD45). CD56 - as a surface molecule expressed on most paediatric tumour cells - was negative in any case. All cell cultures were positive for the MSC markers CD73, CD90, CD105 (Fig. 3a). Dashed line: isotype control; bold line: antibody as indicated. Fig. 3b shows that a dim surface expression of HLA-DR could be found after a four day incubation period with 200 U/ml IFN-γ, which is present in most tumour environments (bold line TStrC in the presence of exogenous IFN-γ, dashed line control). The representative analysis was taken from tumour sample number 3.

TStrC were isolated from eleven paediatric tumours (Table 1). Cells could be successfully propagated under culture conditions used for bone marrow-derived MSC [20]. TStrC were plastic-adherent and displayed a homogenous morphology of a MSC-like triangular shape. All tumour stromal cell cultures homogenously showed cell surface expression of CD73, CD90 and CD105 as well as HLA-ABC in flow cytometric analyses. The cells were negative for the surface expression of CD34, CD45 and HLA-DR (Figure 3; n = 11). However, a four day culture of TStrC in the presence of 200 U/ml gamma-interferon (IFN-γ) resulted in an upregulation HLA-DR on the cell surface, suggesting that TStrC may acquire properties of antigen-presenting cells when migrating to sites of chronic inflammation such as tumours.

**Figure 4:**
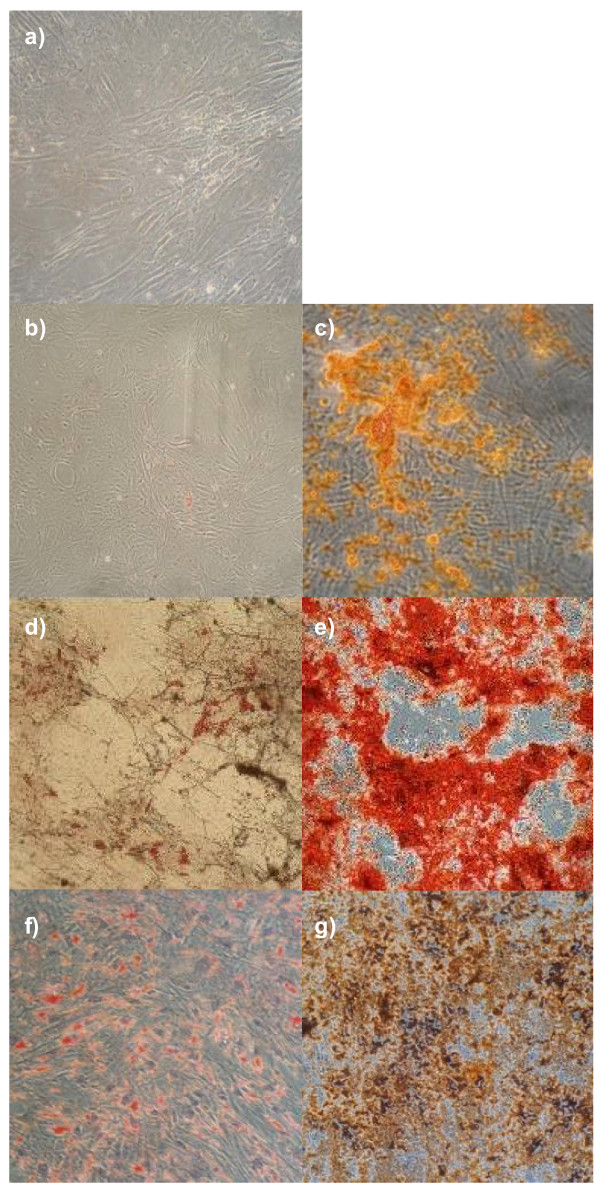
**Morphology and plasticity of TStrC**. a) TStrC, isolated from paediatric tumours, displayed an MSC-like, triangular morphology. b) - g) show differentiation assays toward the adipogenic (b, d, f) and toward the osteogenic (c, e, g) line. Fig. 4b+c: Osteosarcoma TStrC; Fig. 4d+e: Neuroblastoma TStrC; Fig. 4f+g: MSC (control). Osteogenic induction was successful in all 11 TStrC-preparations. However, adipogenic induction yielded poor or no differentiation of TStrC into adipocytes. Representative pictures for each source of stromal cells are shown.

To determine whether the isolated tumour stroma retains not only their phenotypic features of MSC but also their plasticity, we performed differentiation assays toward the osteogenic and adipogenic lineage. MSC were used as positive control. All tumour stroma preparations displayed vigorous osteogenic differentiation potential, verified by Alizarin Red Staining. However, only few cells responded to adipogenic induction (Figure 4; n = 11).

### Anti-proliferative impact of tumour stroma cells on immune effector cells

**Figure 5:**
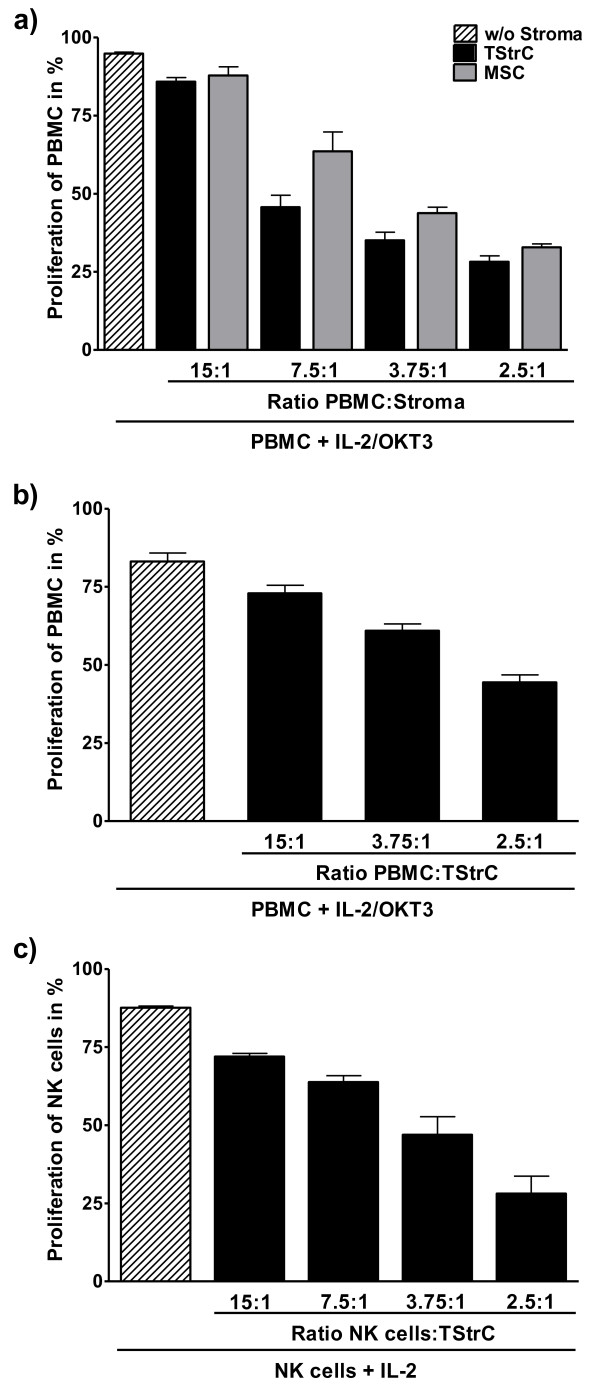
**Anti-proliferative effects of TStrC**. a) CFSE assay to measure the anti-proliferative properties of TStrC. PBMC were stimulated with IL-2 (100 IU/ml) and OKT3 (1 μg/ml). Black bars show the proliferation of PBMC in presence of TStrC, grey bars indicate the proliferation of PBMC in presence of MSC. For each TStrC, a dose dependent inhibition of proliferation was seen (n = 10). b) To mimick the hypoxic tumour-microenvironment, PBMC proliferation assays in presence of TStrC were also conducted under low-oxygen tension (1%). The inhibitory effect of TStrC was maintained under hypoxic conditions (n = 4). c) The anti-proliferative property of TStrC also affected NK cells of different HLA mismatched donors (n = 3). NK cells were isolated using CD56^+ ^magnetic beads and stimulated with 100 IU/ml IL-2.

One important functional property of MSC is their anti-proliferative impact on effector cells of the immune system. In order to test, whether TStrC influence the effector cells similarly, HLA mismatched PBMC were isolated from healthy donors, labelled with CFSE and cocultured with TStrC. For all TStrC cultures examined, proliferation of PBMC was strongly inhibited in a TStrC dose-dependent manner (Figure 5a). The inhibition by TStrC was quantitatively comparable to the anti-proliferative effect exerted by MSC.

The tumour-microenvironment is an area of low oxygen tensions [22]. To determine whether the anti-proliferative impact of TStrC is maintained under hypoxic conditions and may thus contribute to paralysing tumour infiltrating immune cells, the coculture experiments were also performed under 1% oxygen tension. As shown in Figure 5b, the anti-proliferative effect was not abrogated under those conditions, although the inhibition was less potent than under normoxia.

In neuroblastoma and other paediatric tumours with low surface HLA expression, NK cells may play a major role in tumour control by the immune system. Therefore, we focused on NK cell proliferation and found that TStrC strongly inhibit NK cell proliferation as well (Figure 5c).

### Exposition to tumour-stromal cells impairs NK cell cytotoxicity

As cell proliferation in the immune system is only a surrogate marker for functional properties, we next analyzed the cytotoxicity of NK cells. After four days of coculture with TStrC at a ratio of 4:1 (NK cells : TStrC), NK cells were removed, counted and used in cytotoxicity tests against a standard target cell line (K562). NK cells displayed a significantly reduced cytotoxicity (Figure 1a) at E/T ratios of 20:1, 10:1 and 5:1. In order to exclude that the reduced cytotoxic capabilities of cocultured NK cells were due to exhaustion of the effector cells after killing of TStrC during the conditioning period, we analyzed the cytotoxicity of NK cells against TStrC as well. There was only a low cytotoxicity of NK cells against TStrC themselves (Figure 1b). This effect did not lead to an exhaustion of NK cells.

### Tumour stroma alters NK cell receptor expression patterns

To determine, whether the observed decrease in cytotoxicity was caused by an altered expression pattern of activating receptors on the cell surface, NK cells from coculture experiments and their respective control were analyzed for the expression of NKp44, NKp46 (natural cytotoxicity receptors), CD56, CD69 and NKG2A by flow cytometry. Interestingly, IL-2 induced upregulation of the natural cytotoxicity receptors NKp44 and NKp46 was strongly inhibited (p < 0.01) in the presence of TStrC, which may contribute to the decrease in cytotoxicity against K562 (Figure 2). A similar difference was seen in the expression level of CD56, which was reduced after coculture with the tumour stroma. Consistent with the previous results that TStrC render NK cells in an inactive state, the expression levels of the inhibitory receptor NKG2A did not show any difference compared to its respective control. Similarly, the activation marker CD69 was not upregulated, when adding TStrC to the NK cells supporting the notion that the coculture period did not lead to an activation of the cells against the stroma. Taken together, TStrC share several biological features with MSC and may contribute to immune escape strategies, e. g. by interfering with NK cell activation.

## Discussion

The impact of tumour stroma and other components of the tumour microenvironment on the prognosis is well established, e. g. Finak et al. have developed a prognostic score based on the cytogenetic profile of breast cancer stroma [23]. Although the last years have seen a surge in publications highlighting the immunological impact of tumour-microenvironment, additional studies focusing on functional aspects of native human TStrC are sparse [24-26]. In particular the role of TStrC in paediatric neoplasias has not been adressed in current literature. Several source tissues of TStrC have been identified: On the one hand epithelial and endothelial to mesenchymal transition are a major source of stromal cells and on the other hand recruitment of bone marrow-derived MSC into the tumour has been suggested as an origin of TStrC [7,8,25].

In the present study, we demonstrated that isolation of TStrC from paediatric tumours is feasible and that cells can be propagated under standard conditions. Our results indicate that TStrC represent a population similar to bone-marrow derived MSC. Presence of typical MSC markers as well as some overlap in plasticity suggest that human TStrC are related to, but not identical with MSC. Comparative analyses of MSC and TStrC are sparse. However, Zhao et al. have shown that stromal cells derived from prostate cancer express high levels of CD90, but differ in their gene expression pattern from MSC [27].

MSC have been ascribed potent immunomodulatory effects on various cell populations of the immune system. These in vitro findings account for the beneficial effects of MSC in aggressive, immunological processes such as GvHD [28]. Based on the similarities between MSC and TStrC, we asked if there is a direct contribution of TStrC towards a potential immunosuppressive effect of human tumour stroma. This aspect is of critical importance as there are numerous immunotherapeutic approaches currently being implemented in oncological therapy. The presence of immunosuppressive cells may interfere with successful immune surveillance. In analogy to MSC, we found TStrC to exert an anti-proliferative effect on PBMC of healthy donors. This *in vitro *model correlates with the observation that *in vivo *immune cells can be found predominantly in contact to stromal cells within solid tumours without mounting a protective immune response [3]. Thus, recruitment of stromal cells in large numbers such as in pancreatic adenocarcinoma, where TStrC often make up as much as 90% of the total tumour volume, may be part of an immune escape strategy [29].

In our experiments we have shown that TStrC do not only affect the proliferative capacity of immune cells, but also their function. NK cells, which are known to be potent protagonists of the innate tumour defence, display a reduced cytotoxicity after coculture with TStrC. Further analyses revealed that the compromised cytolytic function may be due to downregulation of the activating receptors NKp44 and NKp46, which have been reported to increase the anti-tumour cytotoxicity of NK cells [30,31]. In line with these findings, NK cells derived from native lung tumours have been shown to display an altered receptor expression compared to NK cells from the peripheral blood [3]. Similar observations were also made for renal cell carcinomas [32]. These different tumour models indicate that the tumour microenvironment may compromise the immune reaction to promote cancer development [33,34]. Anatomically, TStrC are located adjacent to blood vessels and establish contact with immune effector cells directly upon extravasation of the latter. Our data indicate that TStrC of paediatric tumours inhibit tumour-infiltrating immune effector cells. More specifically, altered NK cell receptor expression may be due to the tumour stroma rather than the tumour cells themselves.

A recent study on NK cell receptor expression after coculture with bone marrow-derived MSC parallels our findings for TStrC [14,15]. Hence, the two populations of TStrC and MSC not only share several phenotypic features, but may influence effector cell populations such as NK cells in a similar way. The issue in how far immunomodulatory effects of MSC are specific has recently been addressed by Haniffa et al., who have argued that fibroblasts from skin or synovial fluid display anti-proliferative effects on T cells [35]. By contrast, others could not find an impact on immune cell proliferation by different stromal fibroblasts in coculture experiments [11].

Beyond the anti-proliferative effects of tumour stromal cells on PBMC, the impact of the complex tumour microenvironment on effector cell functions is well established in the literature [7,36-38]. The excessive recruitment of stromal cells to tumours and the resulting functional impairment of invading immune cells may thus be part of an immune escape strategy of paediatric tumours. Adding to these in vitro findings, immune effector cells have been found predominantly in the stromal compartment of tumours [3]. These cells are dysfunctional and may not reach the tumour cells themselves.

## Conclusions

Our experiments reveal a crucial role of TStrC derived from paediatric cancers in impeding immune cell functions. Taken together, the tumour stroma represents an important target for successful immunotherapeutic approaches in the clinic.

## Competing interests

The authors declare that they have no competing interests.

## Authors' contributions

PDJ carried out experiments and wrote the manuscript, MV carried out experiments, FG carried out experiments, PM carried out experiments, SAE provided crucial patient samples, TK provided crucial patient samples, RH contributed to the manuscript, IM designed experiments and contributed to the manuscript. All authors have read and approve of the manuscript.

## Pre-publication history

The pre-publication history for this paper can be accessed here:

http://www.biomedcentral.com/1471-2407/10/501/prepub
